# Mentalisation-Based Group Therapy (MBT-G): A Theoretical, Clinical, and Research Manual

**DOI:** 10.1192/pb.bp.115.053116

**Published:** 2017-04

**Authors:** Gwen Adshead

This book presents a challenge for a reviewer because it is both a theoretical text and a technical manual. The technique in question is mentalisation-based group therapy (MBT-G) and one of this book's functions is to assist supervisors of MBT-G in rating therapists on quality of technique and adherence to the MBT-G guidelines. So, at first sight, you might not be drawn to this publication unless you are a group therapist – and trained in MBT-G.

However, I encourage people who are not psychotherapists or trained in MBT to consider this manual as a useful introduction to the concept of mentalising. Mentalisation is an old concept in psychology and refers to our human ability to understand ourselves as agents who make choices and form intentions. This ability includes an understanding and perception of *other* people as having minds that form intentions, which are real and distinct from our own.

All psychiatrists need a valid and reliable model of mind with which to work clinically, and the concept of mentalisation fits the bill. Mentalising capacities are crucial to our social existence, across the lifespan; failure to mentalise successfully is a feature of all mental disorders. The healthy mind is constantly mentalising, with odd lapses in reasoning and dialogue that are neither too severe nor too frequent. When the mind is disordered – through any cause – mentalising fails and immature modes of thinking dominate, often with catastrophic results in terms of social identity and function. The restoration of mentalising then becomes a crucial aspect of all psychiatric treatment.

There are several books on mentalising and mentalisation-based therapy by Karterud's collaborators in the UK (Peter Fonagy and Anthony Bateman) and the USA (Jon Allen). I found this particular book of interest because it approaches mentalising from a philosophical perspective: that of hermeneutics and how we interpret the world. Karterud suggests that the way we interact with and interpret others comes before our experience of our own minds; that the social self is primary in developmental terms. Such a relational approach to mind is a vital complement to models of mind that are either atomistic or mechanical. We have no evidence that the mind works like a machine, but there is growing evidence that the mind is organic and dynamic, responding, developing and evolving in response to the environment – which, for human beings, is the experience of other minds.

MBT is recommended by the National Institute for Health and Care Excellence for the treatment of borderline personality disorder and treatment trials of MBT for antisocial personality disorder are ongoing. But understanding mentalising is a broader objective which all psychiatrists need to achieve. This work is obviously essential reading for trained MBT-G therapists, but it is a useful introduction to mentalising in its own right.

